# Dissociative fugue symptoms in a 28-year-old male Nigerian medical student: a case report

**DOI:** 10.1186/1752-1947-7-143

**Published:** 2013-05-31

**Authors:** Monday N Igwe

**Affiliations:** 1 Department of Psychological Medicine, Federal Teaching Hospital Abakaliki, Ebonyi State, Nigeria

**Keywords:** Dissociative, Fugue, Medical, Student

## Abstract

**Introduction:**

Dissociative fugue is a psychiatric disorder characterized by amnesia coupled with sudden unexpected travel away from the individual’s usual surroundings and denial of all memory of his or her whereabouts during the period of wandering. Dissociative fugue is a rare disorder that is infrequently reported. Before now, no case of it had been reported in a medical student.

**Case presentation:**

This article focuses on the report of a case of dissociative fugue symptoms in a 28-year-old male Nigerian medical student.

**Conclusion:**

The observation in this case report brings to the fore that dissociative fugue is often related to stressful life events and can comorbid with a depressive disorder.

## Introduction

Dissociative fugue, formerly called psychogenic fugue, is one of a group of psychiatric conditions called dissociative disorders. Dissociative disorders are characterized by transient or chronic failures or disruptions of integration of consciousness, memory, perception, identity or emotion. Dissociative disorders include dissociative amnesia, fugue, depersonalization disorder, dissociative identity disorder and dissociative disorder not otherwise specified [1].

People with dissociative fugue temporarily lose their sense of personal identity and impulsively wander away from their homes or places of work [2]. They may travel far distances during the fugue, as far as several thousand miles [2,3]. They may remain in the fugue state for a couple of days, several weeks or even months [2-4]. When individuals return to their pre-dissociative states, events that occurred during the fugue are not remembered [2].

Dissociative fugue is a rare disorder and data available indicate a prevalence of 0.2% in the general population [2,5]. The onset is often in adolescence or early adulthood [6]. Onset is usually sudden [3], and often related to traumatic or stressful life events [2,4,6]. Dissociative fugue has also been noted to be associated with a previous history of child abuse [7] and current severe distress [8]. Other factors predisposing to dissociative reactions include neuropsychological cognitive dysfunctions [9] and genetic factors [10]. Recovery is usually sudden [6] and often complete [2,6], although the fugue state may end gradually in some individuals [3]. However, following recovery there is no amnesia for earlier life events before the dissociative fugue episode [8].

Alcohol, hallucinogens, marijuana, head trauma, brain tumors, dementia, hypertension, manic episode and schizophrenia may cause effects similar to dissociative fugue [3]. It is therefore expedient that a differentiation be made between a dissociative fugue episode and dissociative fugue-like symptoms caused by a medical condition or psychological disorder. Dissociative fugue can comorbid with bipolar disorder, major depressive disorder, schizophrenia, post-traumatic stress disorder, substance-related disorders, panic disorder, anxiety disorders, eating disorders and somatoform disorders [2,3].

Treatment of dissociative fugue is by use of psychotherapy. Attempts are made to elicit stressors preceding the disorder [6], and psycho-education is given to both the patient and family. Efforts are made to reduce stressors that may precipitate another episode. However, if the patient is still in a fugue state, the first concern is to ensure the safety and well-being of the patient. As dissociative fugue is often comorbid with psychiatric disorders, drug treatments may be necessary for the latter.

## Case presentation

The patient is a 28-year old male final year medical student from the South-Eastern region of Nigeria in sub-Saharan Africa. He was declared missing for 10 days prior to presentation because his whereabouts was unknown. He was later seen in a city in South-Western Nigeria, a distance of about 634km from South-Eastern Nigeria where he lived and schooled. Ten days before presentation, while studying in his room alone at night, the patient suddenly saw a full human skeleton reading at the same table with him, sitting at the opposite side. At the same time, the patient claimed he felt unease and quite uncomfortable. He saw the whole room turning with everything inside becoming unstable and unreal. After this he had overwhelming fears and did not know when he left the room. Two days later, he discovered he was with his younger sibling in South-Western Nigeria. The patient had no knowledge of how he made the journey that takes approximately 8 hours by road. He equally could not remember where he slept the night he left his room, how he raised money for the journey or the buses and routes he took. The patient denied all memory of events for the 2 days from when he left his room at the university to the time he suddenly realized he was at his brother’s house, 634km away. The brother, however, reported that the patient appeared unkempt, looked exhausted but was fully conscious and alert on arrival at his house without any assistance.

Prior to this episode, the patient had been under severe economic and academic pressures. The younger brother who paid the patient’s bills had threatened to withdraw his sponsorship because of the patient’s prolonged stay in school beyond the stipulated duration of training occasioned by his repeats of examinations and classes. The patient had been worried that he might also fail in his final qualifying examinations scheduled to be held in 3 months. He subsequently became involved in several religious activities to obviate his perception of impending doom.

The patient admitted to having low mood, loss of interest in usually pleasurable activities and poor appetite. He had lost weight and most often preferred being alone. He had also been feeling weak especially in the morning hours but had managed to grudgingly carry on with the day’s activities. He had suicidal ideation but never attempted suicide. The patient slept poorly at night. His sleep had been marked by early morning wakefulness and waking up not feeling refreshed.

There were no symptoms suggestive of seizure, manic episode, schizophrenia, anxiety or organic disorders. He never drank alcohol or abused any psychoactive substances. The patient denied a history of head trauma or loss of consciousness in the past.

Past medical, psychiatric, family and personal histories revealed no significant findings.

Examination of his mental state revealed a young man who was clean, appropriately dressed and mildly emaciated with poor eye contact. His mood was depressed. He had preceding visual and derealization perceptual disorders. He had no thought disorders. The patient was oriented in time, place and person but had impaired attention and concentration at the time of the examination. Immediate recall, short- and long-term memory were intact. However, there was amnesia for the 2 days he wandered away from school. Judgment and insight were not impaired.

His physical examination was unremarkable. Neurological assessment and basic laboratory testing revealed no significant abnormalities.

An electroencephalogram reported no seizure activity. A computed axial tomography of the brain was not done because the patient lacked resources to pay for it. Also, a test for blood alcohol level and urine drug screening were not done because the hospital had no facilities for the tests.

The Dissociative Experiences Scale (DES) was administered to the patient and he had a score of 50%. The DES is an effective screening instrument for dissociative disorders [11,12].

A diagnosis of dissociative fugue-like syndrome was made with comorbid major depressive episode. He was engaged in psychotherapy by the departmental clinical psychologists and his depression was treated with paroxetine. He responded very well and was able to write his final qualifying examinations 3 months later. He, however, did not pass either of the two subjects examined.

At 6-month follow up, the patient could still not recall events for the 2 days from when he left school to the time he was seen in his brother’s house, 634km away. He reported no further periods of amnesia or wandering away from his place of residence.

## Conclusion

The observation in this case report brings to the fore that dissociative fugue is often related to stressful life events and can occur with a comorbid depressive disorder.

## Consent

Written informed consent was obtained from the patient for publication of this case report. A copy of the written consent is available for review by the Editor-in-Chief of this journal.

## Competing interests

The author declares that he has no competing interests.
